# Waking experience modulates sleep need in mice

**DOI:** 10.1186/s12915-021-00982-w

**Published:** 2021-04-06

**Authors:** Linus Milinski, Simon P. Fisher, Nanyi Cui, Laura E. McKillop, Cristina Blanco-Duque, Gauri Ang, Tomoko Yamagata, David M. Bannerman, Vladyslav V. Vyazovskiy

**Affiliations:** 1 Department of Physiology, Anatomy and Genetics, University of Oxford/Sleep and Circadian Neuroscience Institute, Oxford, UK; 2 Department of Experimental Psychology, University of Oxford, Oxford, UK; 3 Nuffield Department of Clinical Neurosciences, University of Oxford, Oxford, UK

**Keywords:** Mice, EEG, Sleep homeostasis, Behaviour, Wakefulness, Slow-wave activity, Operant behaviour, Running-wheel activity, Exploratory behaviour

## Abstract

**Background:**

Homeostatic regulation of sleep is reflected in the maintenance of a daily balance between sleep and wakefulness. Although numerous internal and external factors can influence sleep, it is unclear whether and to what extent the process that keeps track of time spent awake is determined by the content of the waking experience. We hypothesised that alterations in environmental conditions may elicit different types of wakefulness, which will in turn influence both the capacity to sustain continuous wakefulness as well as the rates of accumulating sleep pressure. To address this, we compared the effects of repetitive behaviours such as voluntary wheel running or performing a simple touchscreen task, with wakefulness dominated by novel object exploration, on sleep timing and EEG slow-wave activity (SWA) during subsequent NREM sleep.

**Results:**

We find that voluntary wheel running is associated with higher wake EEG theta-frequency activity and results in longer wake episodes, as compared with exploratory behaviour; yet, it does not lead to higher levels of EEG SWA during subsequent NREM sleep in either the frontal or occipital derivation. Furthermore, engagement in a touchscreen task, motivated by food reward, results in lower SWA during subsequent NREM sleep in both derivations, as compared to exploratory wakefulness, even though the total duration of wakefulness is similar.

**Conclusion:**

Overall, our study suggests that sleep-wake behaviour is highly flexible within an individual and that the homeostatic processes that keep track of time spent awake are sensitive to the nature of the waking experience. We therefore conclude that sleep dynamics are determined, to a large degree, by the interaction between the organism and the environment.

**Supplementary Information:**

The online version contains supplementary material available at 10.1186/s12915-021-00982-w.

## Background

The duration, timing and intensity of sleep and wakefulness are under strict homeostatic control, and the time spent awake is considered a key determinant of sleep pressure [1]. In addition, most animals partition the 24-h day into consolidated periods of wakefulness and sleep with respect to environmental conditions, such as the light-dark cycle or food availability [2, 3]. Typically, laboratory mice wake up near dark onset (ZT12) and remain awake for a variable duration before entering their first sleep bout. Evidence suggests that the duration of spontaneous wakefulness can be influenced by essential homeostatic needs, motivated behaviours and environmental factors [4]. For example, providing access to running wheels results in an increased capacity to sustain continuous wakefulness [5, 6]. Furthermore, mice have also been shown to have longer waking bouts when food-deprived [2].

Although it has been appreciated that environmental factors play an important role in understanding behaviour in general [7], and sleep in particular [8], surprisingly little is known about the effect of different behaviours elicited by environmental demands on sleep homeostasis. It is well established that sleep following prolonged wakefulness is characterised by elevated levels of electroencephalogram (EEG) spectral power between 0.5–4 Hz (slow-wave activity or SWA), proportional to the duration of preceding waking [9–11]. However, in addition to the time spent awake, evidence suggests that the specific nature of wake behaviours or activities may also influence subsequent sleep. For example, it was established that cortical SWA increases locally in the brain regions that were stimulated during preceding wakefulness [9, 10], suggesting that sleep is regulated in a local, activity-dependent manner [12]. Localised sleep-like activity has also been identified during wakefulness, particularly after prolonged wakefulness or extended training [13–15]. Consistent with the detrimental effects of sleep pressure on cognitive performance [16], these localised changes in cortical activity correlate with performance errors [13, 17], suggesting that the expression of local sleep is incompatible with sustained waking performance. Yet, an important question remains: which aspects of waking behaviour lead to the accumulation of sleep pressure?

Previous studies that investigated the relationship between waking activities and sleep mechanisms often utilised tasks that required precise motor coordination [13, 18, 19] or were designed to trigger neuronal plasticity mechanisms [14, 20–22]. This leaves the possibility that changes in sleep/wake dynamics may not be directly related to the amount of wakefulness per se but could instead arise as a result of the cognitive and/or attentional load of the behavioural tasks performed during waking and their related neural activity [22, 23]. Arguably, engaging in well-practised, less cognitively demanding behaviour while awake may have a very different effect on the accumulation of sleep pressure.

There is indeed evidence that sufficient training in a task may allow performance to become stereotypical and to require sustained activity only within restricted brain networks [24]. Execution of well-trained tasks can become independent of the primary motor cortex [25] or, as has been shown in song birds, independent of the forebrain [26]. Thus, it is possible that a task involving merely the execution of a simple motor-sequence rather than constantly adapting behaviour may be sustained even during localised changes in cortical activity or ‘local sleep’ in some cortical areas. Since evidence suggests that local cortical neuronal activities represent an important factor in governing global sleep homeostasis [27], engagement in well-trained behaviours may therefore result in an attenuated build-up of sleep need during waking. Consistent with this notion, it has been shown that stereotypic, repetitive wheel running is associated with a substantial prolongation of wake periods [11], yet this does not lead to an increase in cortical excitability, which was previously associated with increased sleep pressure [5, 28–30]. Although the neurophysiological mechanisms underlying the increased capacity to sustain spontaneous wakefulness are unclear, we previously proposed that wakefulness dominated by simple, stereotypic behaviours may be associated with reduced sleep need [5].

Here, we directly investigated the effects of two types of repetitive, stereotypic behaviour on sleep timing and EEG SWA during subsequent sleep. Specifically, we examined the effects of spontaneous running in a running wheel and voluntary performance in a well-learned, simple operant task involving food rewards. Both behaviours were compared to a condition in which animals were sleep deprived by providing novel objects to induce active, exploratory behaviour. We hypothesised that the nature of the waking behaviours has important influences on subsequent sleep, above and beyond its duration.

## Results

### Experiment 1: Voluntary wheel running increases the latency to sleep onset

**Fig. 1:**
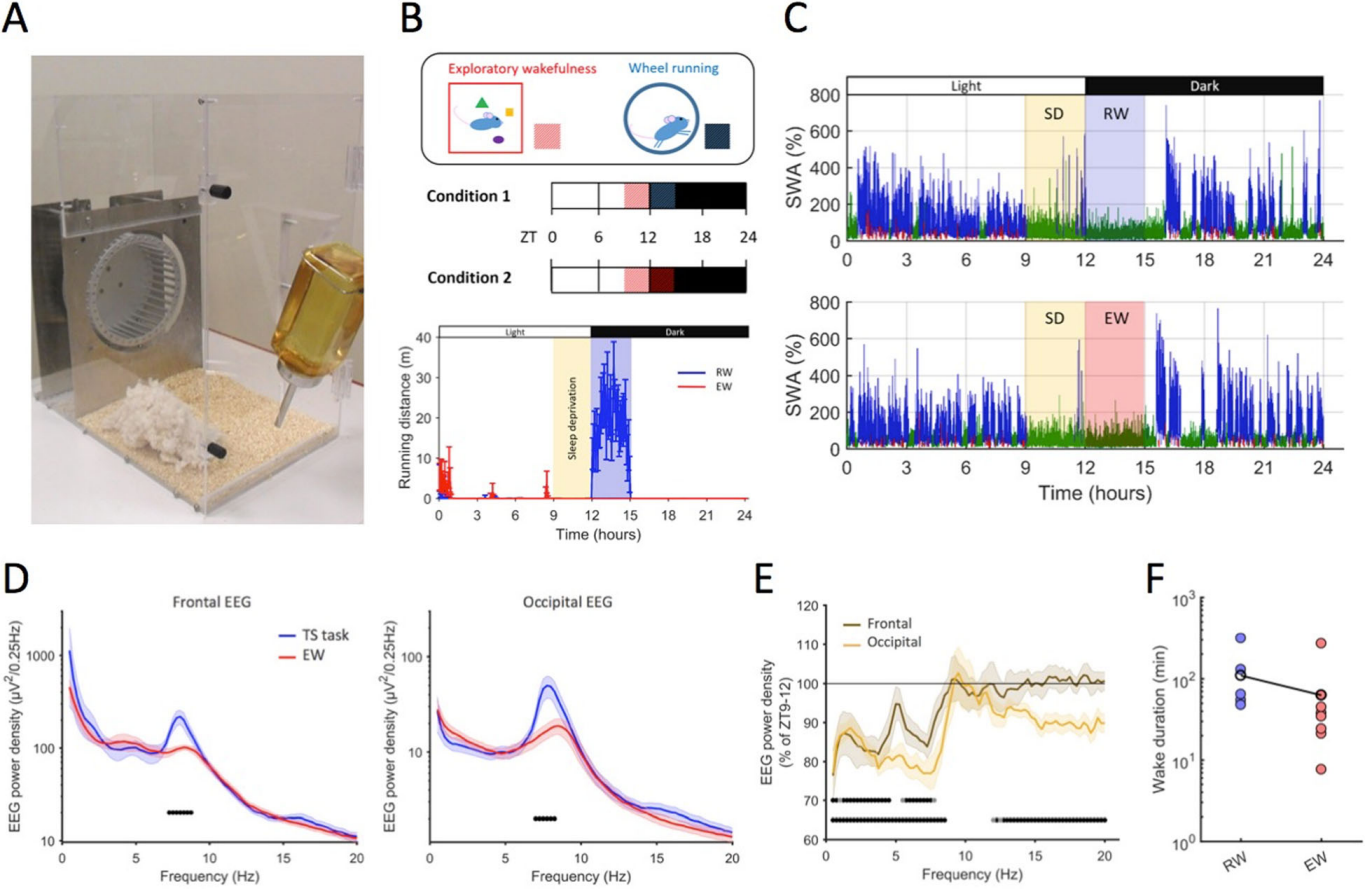
The effect of voluntary wheel running on wakefulness and sleep. **a** Photograph of a mouse home cage, fitted with a running wheel. EEG recordings were acquired continuously in the freely moving animals while in the home cage. **b** Top: outline of the experiment. In both experimental conditions, animals were first kept awake by providing novel objects for the last 3 h of the light period (ZT9–12). Subsequently, between ZT12–15, mice had access to running wheels (RW condition) or were kept awake by providing novel objects without running wheel access (EW condition). EEG recordings were acquired and analysed over the entire 24-h period between ZT0–24 in either condition. Bottom: time course of RW activity during the experiment, shown in 5-min bins. Note that the first 3 h of the dark period (ZT12–15) is dominated by spontaneous wheel running in the RW condition only. **c** Hypnogram of a representative mouse during the two experimental conditions, RW (top) and EW (bottom). The plots depict colour-coded EEG slow-wave activity (wakefulness: green, NREM: blue, REM sleep: red) with a 4-s epoch resolution, shown as % of the mean SWA over the 24-h period. **d** Wake EEG spectra during the wheel running ‘RW’ and the exploratory wakefulness ‘EW’ condition. Mean values, SEM. Horizontal bars depict frequency bins where differences between the RW and EW spectra were statistically significant (*p* < 0.05). **e** Relative wake EEG spectra during the exploratory wakefulness (ZT12–15) for frontal and occipital EEG derivations, expressed as percentage of EEG power during wake between ZT9–12 (100%). Significant differences from ZT9–12 are shown as horizontal lines (top: frontal, bottom: occipital). **f** Latency to the first consolidated sleep episode > 1 min (RW, 109.6 ± 42.6; EW, 62.9 ± 30.1 min, *p* = 0.04, Wilcoxon rank sum test). The dots indicate individual mice, black line connects dots signifying average values across animals

In previous experiments, the effects of running wheel activity on sleep were studied in mice with unrestricted access to wheels [5, 6]. Here, we compared wakefulness dominated by wheel running with wakefulness dominated by exploratory behaviour in which animals were provided with novel objects. All animals were well accustomed to running and had unrestricted access to running wheels (Fig. 1a, b) prior to ZT9 on each experimental day. As expected, during the light period (typical rest phase of mice) before the experiment commenced, all animals showed negligible amounts of running (Fig. 1b) and a typical amount and distribution of sleep (data of a representative mouse are shown in Fig. 1c).

Mice were tested in two experimental conditions on separate days (Fig. 1b, top). On both experimental days, all animals were kept awake using novel objects for 3 h starting from ZT9 until the beginning of the dark period at ZT12 (Fig. 1b, c) while running wheels were blocked to prevent their use. At the dark onset, in one condition, mice were sleep-deprived for an additional 3 h, again by providing novel objects to elicit predominantly exploratory behaviour (exploratory wakefulness (EW)). In the second condition, mice were instead left undisturbed and given unrestricted access to the running wheel (RW), which all animals used extensively (on average 1552.6 ± 251 wheel revolutions performed during the 3-h interval, which corresponded to 669.7 ± 110.4 m total distance covered, Fig. 1b, bottom). No novel objects were provided during this time, and the animals stayed awake spontaneously. Importantly, the total amount of sleep the animals obtained between ZT0 and ZT9, prior to SD, was identical between the two conditions (RW, 6.15 ± 0.3; EW, 6.12 ± 0.2 h, *p* = 1, Wilcoxon rank sum test), as was the amount of sleep that occurred during 3-h SD between ZT9–12 (RW, 12.4 ± 3.8; EW, 10.5 ± 2.1 min, *p* = 0.75, Wilcoxon rank sum test) or during the following 3-h experimental manipulation between ZT12–ZT15 (RW, 1.5 ± 0.8; EW, 4.6 ± 2.2 min, *p* = 0.49, Wilcoxon rank sum test). At ZT15, 3 h after the dark onset, all animals were then left undisturbed without wheel access for the rest of the dark period and could sleep ad libitum.

To investigate whether wakefulness during the ZT12–15 was qualitatively different between RW and EW conditions, we calculated the average EEG power spectra during wakefulness (Fig. 1d). As expected, we observed markedly higher spectral values in the theta frequency range associated with running, both in the frontal and in the occipital derivations. In the EW group, wake EEG spectral power in low frequencies (0.5–7.5 Hz) was significantly decreased during the ZT12–15 vs the ZT9–12 interval in both derivations (Fig. 1e), which is in accordance with high behavioural activity, although the mice had spent 3 h awake prior to the dark onset. While there was no strong association between running wheel revolutions and sleep latency in the RW group (Pearson correlation *r* = − 0.22, *p* = 0.67), we found that the overall latency to sleep was significantly longer, by on average 47 min, in the RW condition as compared to the EW condition (RW, 109.6 ± 42.6; EW, 62.9 ± 30.1 min, *p* = 0.04, Wilcoxon rank sum test, Fig. 1f). This observation was surprising, given the notion that theta activity corresponds to an awake state associated with a faster build-up of sleep pressure [31].

EEG power density in the fast delta and slow theta frequency (2–6 Hz) ranges during wakefulness is thought to reflect the intrusion of “local sleep” into wakefulness and consistently increases when sleep pressure is high [13–15]. We hypothesised that if wakefulness dominated by exploratory behaviour and running wheel activity is associated with a differential build-up of sleep pressure, then EEG power in this frequency range will be different between the two conditions. Interestingly, we observed that during the first 15-min interval after the end of the RW/EW procedure, when the animals were left undisturbed, EEG power density in the fast delta/slow theta (2.5–7 Hz) frequency range was decreased in both conditions, relative to pre-RW/EW levels, but less so in the EW group (Additional file 1: Figure S1).

**Fig. 2:**
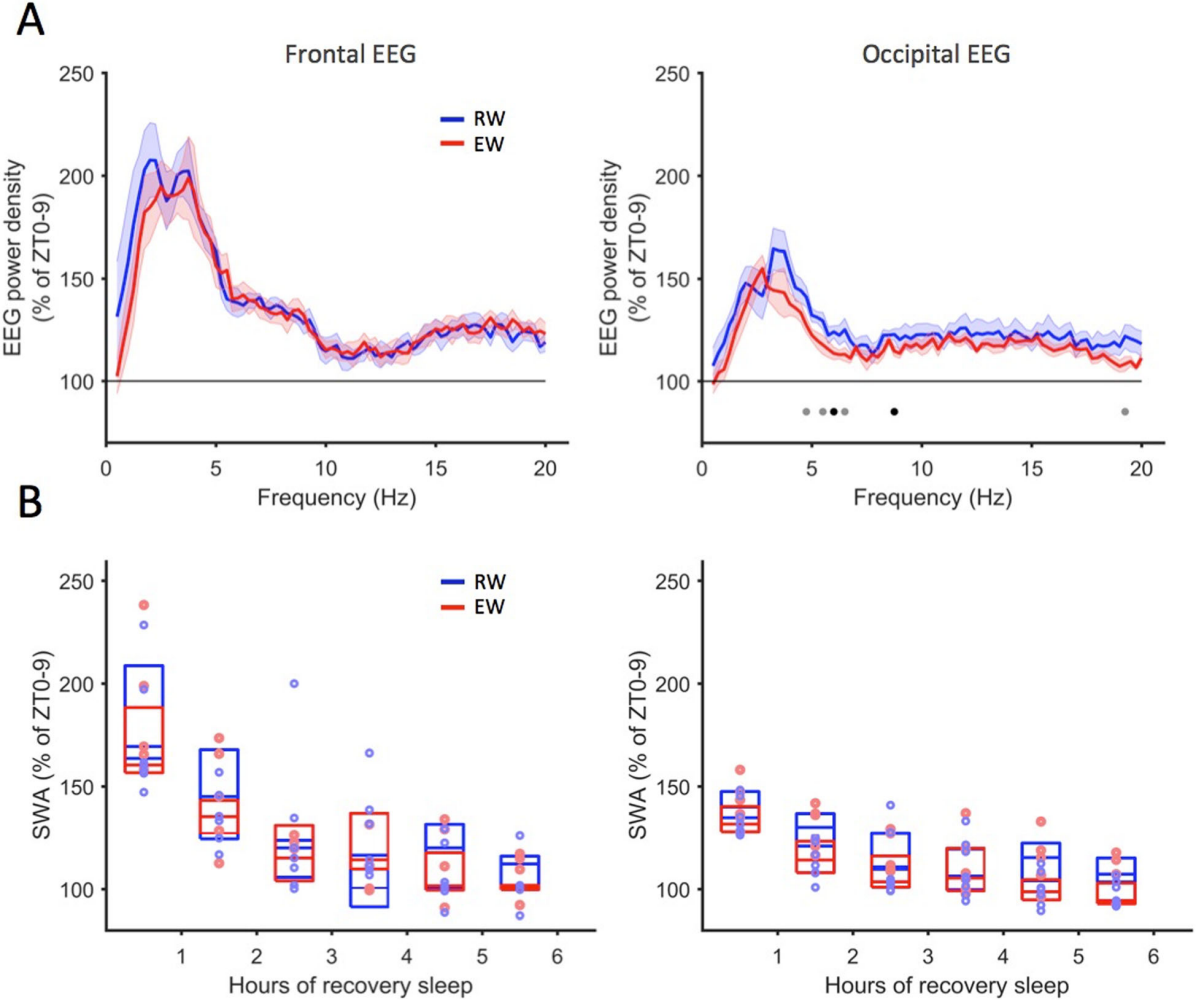
The effect of voluntary wheel running on sleep EEG spectra and SWA. **a** Average NREM EEG spectra of the first hour of recovery sleep after the wheel running (RW) and exploratory wakefulness (EW) conditions. Shaded areas depict standard errors of the mean. Horizontal lines denote frequency bins where EEG power was different between RW and EW conditions (black: *p* < 0.05, grey: *p* < 0.1, Wilcoxon rank sum tests). **b** Cortical EEG slow-wave activity (0.5–4 Hz EEG band) during recovery NREM sleep after RW and EW. Points represent average SWA of individual animals shown in hourly bins, depicted as percentage of average SWA of preceding baseline day (frontal EEG: mixed-model ANOVA (factors hour, condition) on log-transformed data showed statistically significant effect of hour (*F* (5, 40)=27.97, *p* < 0.0001), but no effect of condition (*F* (1, 8)< 1, *p* = 1) and no statistically significant interaction (F (5, 40)=0.93, *p* = 0.47)). Occipital EEG: data were not normally distributed, yet neither transformed nor original data yielded a statistically significant effect of condition or a significant interaction between hour and condition (mixed-model ANOVA on log-transformed data: statistically significant effect of hour (*F* (5, 50)=31.6, *p* < 0.0001) yet no effect of experimental condition (*F* (1, 10)=2.6, *p* = 0.14) or interaction (*F* (5, 50)=1.2, *p* = 0.33); the same test on non-transformed data: significant effect of hour (*F* (5, 50)=33.7, *p* < 0.0001), no significant effect of experimental condition (*F* (1, 10)=1.4, *p* = 0.15) and no significant interaction (*F* (5, 50)=1.13, *p* = 0.36))

There was no significant difference in the overall amount of sleep after RW and EW conditions (Additional file 1: Figure S2), and the total amount of sleep during the first 1-h after sleep onset was similar (RW, 36.1 ± 4.9 min; EW, 37.6 ± 3.8 min, *p* = 0.75, Wilcoxon rank sum test). No major difference between RW and EW conditions was found in EEG power spectra during the initial NREM sleep in either derivation (Fig. 2a), although consistent with the well-known frontal predominance of the EEG SWA rebound [32, 33], the increase was greater in the frontal EEG in both RW and EW conditions. The time course of EEG SWA was generally similar between RW and EW conditions (Fig. 2b).

Sleep fragmentation represents a sensitive measure of sleep depth or sleep intensity, and therefore, we next calculated the number and duration of sleep episodes during sleep after RW and EW conditions. We found that NREM episode incidence during the first 3 h from sleep onset was similar between RW and EW conditions (14.2 ± 2.0 and 12.3 ± 0.8, *p* = 0.29), and there was no difference in the average duration of episodes (6.1 ± 0.9 and 5.6 ± 0.5 min, *p* = 0.75). Similarly, REM sleep episodes occurred at a similar frequency after RW and EW experiments (11.2 ± 1.4 and 8.9 ± 1.2, after RW and EW, respectively, *p* = 0.36), and their average duration was not altered significantly (1.1 ± 0.2 and 1.2 ± 0.1 min, *p* = 0.35). Finally, the incidence of brief awakenings per unit of sleep time was virtually identical after RW and EW conditions (11.3 ± 2.8 vs 11.5 ± 1.2 per hour of sleep after RW and EW, respectively, *p* = 0.85).

Thus, wakefulness dominated by wheel running was associated with high theta frequency power, yet resulted in a delayed sleep onset as compared to wakefulness dominated by exploratory behaviour, without any further changes in sleep amount, fragmentation or SWA.

### Experiment 2: Performance in a well-trained touchscreen task reduces the build-up of sleep pressure

Similar to experiment 1, this paradigm was designed to address the effect of a simple, well-trained behaviour vs exploratory wake behaviour on subsequent sleep. However, in contrast to the RW experiment, in which a certain amount of wakefulness (6 h) was enforced, this experiment was specifically designed to encourage voluntary wakefulness while animals performed a simple touchscreen task. The duration of the exploratory wakefulness condition was then time matched to the duration of the touchscreen task.

**Fig. 3:**
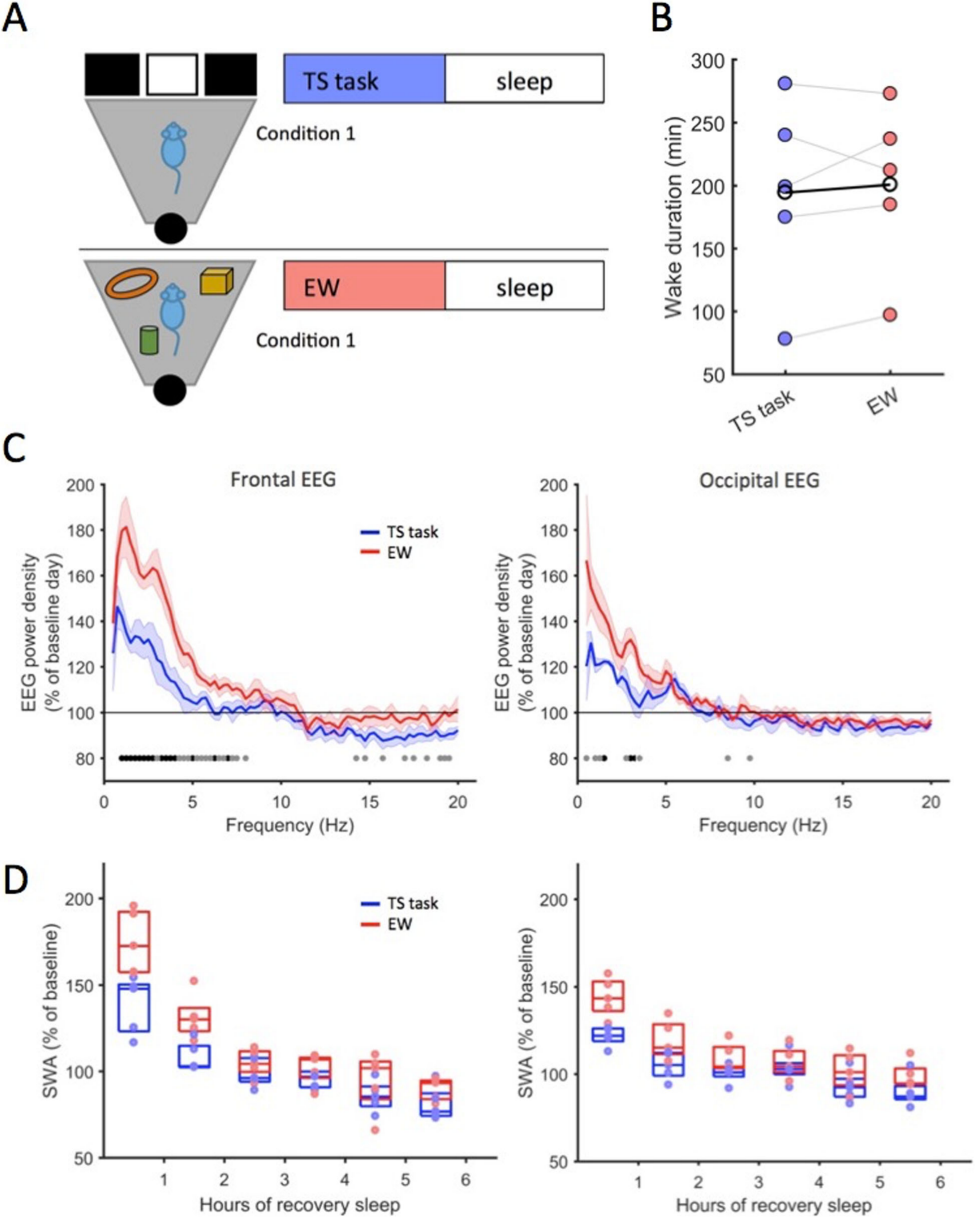
Effect of operant behaviour on sleep and NREM EEG spectra. **a** Outline of the experiment. In condition 1, mice perform the TS task for ad libitum duration from the light onset. In condition 2 (EW), mice are kept awake with novel objects for the corresponding duration to the TS performance. **b** Total wake durations from before the respective experimental manipulation (TS or EW) until sleep onset afterwards. Points depict individual animal averages, black line depicts the mean across animals (*p* = 0.6, n.s., Wilcoxon signed rank test). **c** Average NREM EEG spectra of the first hour of recovery sleep after TS and EW conditions. Shaded areas depict standard errors of the mean. Horizontal lines denote frequency bins where EEG power was different between TS and EW conditions (black: *p* < 0.05, grey: *p* < 0.1, Wilcoxon rank sum tests). **d** EEG slow-wave activity (0.5–4 Hz EEG band) during recovery NREM sleep after TS and EW. Dots represent the average SWA of individual animals shown in hourly bins, depicted as percentage of average SWA of preceding baseline day (frontal EEG: repeated measures ANOVA (factors hour, condition) on log-transformed data revealed statistically significant effect of hour (*F* (7, 28)=32.6, *p* < 0.0001), and a significant interaction between condition and hour (*F* (7, 28)=4.7, *p* < 0.01)). Tukey post hoc tests revealed a significant difference between TS and EW for hours 1 and 2 (hours 1–6: *p* = 0.0009, 0.003, 0.45 0.29, 0.42, 0.22). Occipital EEG: significant effect of hour (*F* (7, 28)=19.08, *p* < 0.0001), significant interaction between hour and condition (*F* (7, 28)=4.75, *p* < 0.01) and a significant effect of condition (*F* (1, 4)=15.09, *p* < 0.05). Tukey post hoc testing revealed significant differences between TS and EW as follows: hours 1–6: *p* = 0.0005, 0.002, 0.1, 0.07, 0.09, 0.009


Prior to the experimental day, all mice (*n* = 5) were trained in an operant touchscreen (TS) task in a Bussey-Saksida Touch Screen chamber (Fig. 3a, Additional file 2: Video V1, Additional file 1: Figure S3A) for a minimum of 16 days. Animals showed a marked increase in the amount of trials completed over the course of training (Additional file 1: Figure S3B) with 31 ± 5 trials per session within the initial 5 days and 148 ± 18 trials during the final 5 days of training. After having completed at least 100 trials in a single session, animals performed the main experimental paradigm in which the mice were placed in the touchscreen chamber at light onset (ZT0) and allowed to perform the task at will. Once the criteria for continuous, spontaneous disengagement from the task were met (see the “Methods” section, on average after 2.5 h), the animals were transferred back to their home cages and undisturbed sleep recordings were obtained until the end of the subsequent dark period (ZT24). Three to four days later, at the light onset, the animals were again transferred into the TS operant chambers at ZT0 but instead of performing in the touchscreen task were kept awake by using novel objects (exploratory wakefulness (EW)) for the same duration as they had each previously performed the TS task.

**Additional file 2: Video V1.** Performance in the touch screen (TS) task. A trial consists of nose poking a reward tray (bottom of the screen), followed by a single touch to an illuminated screen located in the centre of the opposite wall of the chamber. During the training stage, completion of every trial is rewarded by liquid food reward administered into the reward tray. During the testing stage every third trial is rewarded. Nose poking the reward tray initiates the next trial.

During unrestricted TS task performance, which lasted on average 2.5 h (individual values 1, 2, 3, 3 and 3.5 h, *n* = 5), the animals completed 156, 307, 544, 871 and 732 trials, respectively (Additional file 1: Figure S3C). As intended, the overall waking time (wake onset before the experimental manipulation to sleep onset afterwards) was nearly identical between the TS task and EW days (195 ± 34 min and 201 ± 30 min respectively, *p* = 0.63, Fig. 3b), as was the total amount of sleep the animals obtained during the 12 h dark period prior to the TS and EW days (TS 328.4 ± 14.1 min, EW 304.1 ± 19.6 min, *p* = 0.44), or during the preceding 12 h light period (TS 391.5 ± 22.1 min, EW 414 ± 19.2 min, *p* = 0.06, paired Wilcoxon signed rank test). NREM sleep fragmentation in the first 3 h of recovery sleep was not significantly different between experimental days (episode numbers TS 16.8 ± 0.49, EW 17.8 ± 0.86, *p* = 0.56), neither was the incidence of brief awakenings per hour of sleep (TS 12.2 ± 0.49, EW 16.0 ± 1.68, *p* = 0.16). Only REM episodes were slightly more frequent after the EW condition (TS 7.8 ± 0.2, EW 9.0 ± 0.32, *p* = 0.04). The average duration of sleep episodes was similar after TS and EW conditions for both NREM sleep (TS 7.6 ± 0.66 min, EW 6.8 ± 0.61 min, *p* = 0.35) and REM sleep (TS 1.5 ± 0.15 min, EW 1.4 ± 0.14 min, *p* = 0.34).

While sleep latencies and distribution were overall similar after TS and EW wakefulness, we observed marked differences in the EEG power spectra (Fig. 3c). Specifically, in both the frontal and the occipital derivation, EEG power density in the SWA frequency range during the first 1-h interval was substantially lower after the TS task, as compared to the EW condition. To investigate the temporal dynamics of this effect, we next calculated the time course of SWA during the first 6 h following the TS/EW wakefulness. In the frontal derivation, we found that EEG SWA was significantly lower during the initial 2 h of recovery sleep in the TS condition as compared to after EW (interaction between factors ‘hour’ and ‘condition’ in two-way repeated measures ANOVA on log-transformed data: *F* (7, 28)=4.7, *p* < 0.01; first hour EW 174.8 ± 2.3% vs TS 138.6 ± 2.1%, *p* < 0.001, second hour EW 131.3 ± 1.6% vs TS 108.3 ± 1.1%, *p* < 0.01, Tukey post hoc test, Fig. 3d). The reduced early NREM SWA after the TS as compared to the EW condition was evident in every individual animal (Additional file 1: Figure S4), and the effect sizes of group-level comparisons were large during the first 2 h (Cohen’s *d*: hour 1 = 4.0, hour 2 = 2.7).

Although sleep pressure is most strongly reflected in frontal SWA we also observed a similar effect of waking condition (TS vs EW) on the occipital EEG derivation (Fig. 3c, d). We found an interaction between the factors ‘hour’ and ‘condition’ (*F* (7, 28)=4.75, *p* < 0.01, repeated measures ANOVA on log-transformed data), and, similarly to the frontal EEG derivation, SWA in the initial 2 h of recovery sleep after touchscreen task performance (TS) was lower than after exploratory wakefulness (EW) (first hour EW 144 ± 5% vs TS 121.7 ± 2.4%, *p* < 0.001, second hour EW 119.4 ± 4.9% vs TS 104.9 ± 3.5%, *p* < 0.01 and 6th hour EW 99.2 ± 3.4% vs ST 89.0 ± 4%, *p* < 0.05, Tukey post hoc test).

To estimate the effect of EW and TS conditions on wakefulness, we calculated wake EEG spectra immediately preceding the TS task as compared to wake EEG spectra immediately after the task, before the animals fell asleep, but observed only a weak tendency to higher power after EW (Additional file 1: Figure S5), an effect much more pronounced during early recovery sleep (Fig. 3d).

The similar modulation of frontal and occipital SWA after EW, as compared to after TS performance, suggests that waking experience can affect cortical SWA in a global manner. Voluntary engagement in a well-trained operant task reduces the build-up of sleep pressure when compared to wake dominated by exploratory behaviours. Overall, our data indicate that environmental conditions have a major effect on wake behaviours, which, in turn, influence subsequent sleep.

## Discussion

Here, we used two complementary experimental approaches to address whether the nature of wake behaviour makes an important contribution towards the dynamics of sleep pressure, rather than this being determined only by wake duration. First, we show that wakefulness dominated by wheel running behaviour results in a prolongation of spontaneous wakefulness without producing an increase of EEG SWA during subsequent sleep as compared to EW. In addition, we demonstrate that voluntary engagement in an operant task that requires repetitive, well-practised behaviour only, reduces the levels of SWA during subsequent sleep, as compared to exploratory behaviour, despite the total wake duration being similar. We conclude that wakefulness dominated by performance in a well-trained task may correspond to ‘waking with a lower cost’.

Although both types of repetitive, well-practised behaviour investigated in this study (running wheel activity and touch screen operant task performance) were associated with a reduced accumulation of sleep pressure, their effects manifested in a different manner (i.e. prolonged wakefulness or decreased SWA during sleep). This was likely determined by the experimental design, including the time of day when the experiments were performed and the specific environmental manipulation(s) used. In the running wheel experiment, the animals were kept awake either by providing novel objects while the running wheel was blocked or by providing unrestricted access to the wheel, which the animals used extensively. This kind of intervention was expected to be most efficient in the dark phase, which is the habitual wake period in mice. It allowed us to assess how long it would take for the animals to fall asleep spontaneously following exploratory behaviour or wheel running. We capitalised on the notion that wakefulness would be sustained until sleep pressure reaches a certain upper threshold thereby initiating sleep. Consistent with our prediction, we found that the animals stayed awake significantly longer after wakefulness dominated by wheel running, as compared to exploratory wakefulness, yet the levels of SWA during subsequent sleep were identical between the conditions. On the contrary, in the touchscreen experiment, the animals stayed awake during the light phase by voluntarily performing a well-practised, appetitively motivated, operant task (TS task). This experiment was performed during the light phase as this is the habitual sleep period of laboratory mice, and so the total wake duration could be closely matched between the TS task condition and novel object EW condition. This allowed us to assess the levels of SWA during subsequent sleep after a similar time spent awake but with very different waking experiences. As predicted, we found that SWA, which is an established measure of the homeostatic sleep pressure [1, 34], was significantly higher after EW as compared to TS performance.

It is typically assumed that while the total sleep time differs greatly across the animal kingdom [35], it is relatively stable within a species, suggesting that it is, at least to some extent, genetically determined [36]. However, more recent evidence reveals a previously underappreciated flexibility with respect to the timing and duration of sleep [2, 5, 8, 37, 38], suggesting that extrinsic factors also play a major role. Here, we provide evidence to support the notion that the nature of waking behaviours in laboratory conditions affects the dynamics of sleep, which in turn provides novel insights into the neurobiological substrates of sleep homeostasis [1]. Specifically, in this study, we altered the type of wake behaviour by manipulating environmental conditions, which then affected the capacity to sustain continuous wakefulness or sleep intensity during subsequent sleep, depending on the time of day and task used.

Active wakefulness associated with higher neuronal activity [27] or EEG theta power [31] has previously been associated with increased homeostatic sleep pressure. The underlying neurobiological substrate of this association remains to be determined, yet its clear implication is that some behaviours may be associated with faster accumulation of sleep need. In support of this, GluA1 AMPA receptor knockout mice have been shown to exhibit elevated SWA during sleep [39] and increased EEG theta power in both the hippocampus and prefrontal cortex (and increased coherence of this theta activity across regions), which is tied specifically to elevated levels of exploratory attention in these mice [40]. Our data suggest, however, that theta activity can be dissociated from the sleep homeostatic process, for example, in conditions when it is related to the performance of a simple, repetitive behaviour, such as wheel running. These findings are consistent with an earlier observation that providing mice with running wheels leads to a major extension of spontaneous wake periods without producing an excess SWA during subsequent sleep [6].

There are other examples when certain behavioural activities may lead to reduced or even negligible build-up of sleep need during waking. For example, wakefulness during food anticipatory activity in mice has been associated with decreased EEG SWA during sleep [2]. With regard to the present data, there is the possibility that wakefulness dominated by repetitive or well-trained motor sequences may be the basis of a reduced build-up of sleep need. It has been previously suggested that, at least in some brain regions, neuronal activity during stereotypic running is functionally closer to sleep than to an awake state dominated by goal-directed purposeful behaviour [5]. This notion is consistent with the finding that several animal species can reduce their sleep time dramatically when prolonged wakefulness is ecologically relevant or not optional, such as in some species of birds during migration [41, 42] or in marine mammals [43–45]. It is likely, therefore, that substantial differences in the amount of sleep between species and at the individual level may be related to qualitative differences in waking behaviour, in addition to the need to satisfy fundamental biological drives such as reproduction [37].

The lack of progress in establishing direct links between states of vigilance, overt behaviour and specific EEG activities has been acknowledged for some time. Major advancements over the last few decades have led to the appreciation that waking and sleep are highly dynamic states, which form a continuum, rather than representing distinct all-or-none conditions. While mixed states have been traditionally viewed as a maladaptive phenomenon that leads to impaired performance, the possibility remains that these states may have an adaptive value. For example, when sleep-like activity occurs during wakefulness in brain areas not directly involved in task execution, essential behavioural performance could be maintained for extensive periods of time. This has previously been shown in great frigate birds who stay in the air for many days while, intermittently, entering mostly unihemispheric sleep, correlated with gliding flight but not manoeuvring [41, 42]. Here, we propose that the notion of maintaining certain wake behaviours during mixed states might not be specific to certain species but could be a wider phenomenon. Most likely candidates for waking behaviours compatible with mixed states are those that may not require coherent activation across large cortical networks, such as execution of well-trained motor sequences, which have been shown to persist even with substantial cortical lesions [25, 26].

There are several acknowledged limitations of our study that merit discussion. First, the number of animals used is relatively low, and future studies may be required to replicate our findings, especially where a lack of effect is claimed. Importantly, however, the key result of reduced SWA during sleep after the touchscreen task as compared to after exploratory wakefulness was observed in every individual mouse, resulting in a very large effect size. Second, it remains to be addressed why wake experience had effects on some characteristics of sleep, such as SWA or sleep latency, and not others, such as sleep fragmentation. While it can be expected that SWA or sleep latency is directly proportional to preceding spontaneous wake duration or sleep deprivation [11, 46–48], this is not always the case. We have shown earlier that the levels of SWA during NREM sleep do not increase linearly with the increase in wake time, but saturate after a few hours [6]. It remains to be determined whether the effects of preceding sleep-wake history and experience differ with respect to their effects on other metrics, such as the number of brief awakenings or sleep episode duration. Our current study did not provide a strong evidence for that, but future studies, utilising larger sample sizes and ideally a “dose dependence” where both wake experience and wake duration are varied independently may be more informative in this regard. Third, the effects of stress cannot be entirely ruled out when different types of waking behaviour on subsequent sleep are compared [49]. For example, previous studies indicated the role of social stress [50] in sleep SWA, and some studies suggest that sleep deprivation by providing novel objects or “gentle handling” is associated with higher levels of corticosterone, although these data are not entirely consistent [51, 52]. Importantly, it was shown that surgical removal of adrenal glands had a major effect on sleep-deprivation induced changes in cerebral gene expression without any effects on sleep SWA [53]. Finally, an important caveat remains that the effects we observed in the TS condition (experiment 2) and, especially, in the running wheel condition (experiment 1) as compared to exploratory wakefulness may be, at least to some extent, be accounted for by the different amount or intensity of physical activity. Previous human studies have shown that exercise can promote sleep [54–56], and acute exercise before bedtime can increase sleep amount [57, 58], but could also interfere with deep NREM sleep in the first hours of sleep [59]. We did not observe any disruption in NREM sleep after our manipulations, but additional studies are necessary to further investigate the role of physical activity associated with different behaviours on sleep and sleep homeostasis.

## Conclusion

We found that wakefulness dominated by the execution of a repetitive, well-trained behaviour represents a state associated with a slow build-up of sleep pressure. Our data suggest a considerable flexibility in sleep-wake architecture and the dynamics of the sleep homeostatic process, which are revealed when altered environmental conditions result in fundamental shifts of wake behaviour. Furthermore, we posit that wake duration and EEG slow-wave activity during sleep may be dissociated, suggesting a differential contribution of intrinsic and extrinsic factors in their respective control.

## Methods

### Experimental animals

Adult male C57BL/6J mice were used in this study (total *n* = 8 and *n* = 5 in the first and the second experiment respectively, see below). The animals were individually housed in custom-made clear Plexiglas cages (20.3 × 32 × 35 cm) with free access to a running wheel (RW). Cages were housed in ventilated, sound-attenuated Faraday chambers (Campden Instruments, Loughborough, UK, two cages per chamber) under a standard 12:12-h light-dark cycle (lights on 0900, ZT0, light levels ~ 120–180 lx). In experiment 1, food and water were available ad libitum, while in experiment 2, moderate food restriction (animals were kept at 85–90% of their average free-feeding weight) was utilised to motivate performance. Room temperature and relative humidity were maintained at 22 ± 1 °C and 50 ± 20%, respectively. Mice were habituated to both the cage and the cables connected to the cranial implant for a minimum of four days prior to recording. All procedures conformed to the Animal (Scientific Procedures) Act 1986 and were performed under a UK Home Office Project Licence in accordance with institutional guidelines. All efforts were made to minimise the number of animals used in this study in line with core 3Rs principles.

### Surgical procedures and electrode configuration

Surgical procedures were carried out using aseptic techniques under isoflurane anaesthesia (3–5% induction, 1–2% maintenance). During surgery, animals were head-fixed using a stereotaxic frame (David Kopf Instruments, CA, USA), and liquid gel (Viscotears, Alcon Laboratories Ltd., UK) was applied to protect the eyes. Metacam (1–2 mg/kg, s.c., Boehringer Ingelheim Ltd., UK) was administered preoperatively. EEG screws were placed in the frontal (motor area, M1, AP + 2 mm, ML + 2 mm) and occipital (visual area, V1, AP − 3.5–4 mm, ML + 2.5 mm) cortical regions. A reference screw electrode was placed above the cerebellum. EEG screws were soldered (prior to implantation) to custom-made headmounts (Pinnacle Technology Inc. Lawrence, USA), and all screws and wires were secured to the skull using dental acrylic. Two single-stranded, stainless steel wires were inserted on either side of the nuchal muscle to record electromyogram (EMG). At the end of the surgery, animals were administered saline (0.1 ml/20 g body weight, s.c.). Thermal support was provided throughout the surgery and during recovery afterwards. Metacam (1–2 mg/kg) was orally administered for at least 3 days after surgery. A minimum 2-week recovery period was permitted prior to cabling the animals.

### Signal processing and analysis

Data acquisition was performed using a Multichannel Neurophysiology Recording System (TDT, Alachua FL, USA). Cortical EEG was recorded from frontal and occipital derivations. EEG/EMG data were filtered between 0.1 and 100 Hz, amplified (PZ5 NeuroDigitizer pre-amplifier, TDT, Alachua, FL, USA) and stored on a local computer at a sampling rate of 257 Hz (experiment 1) or 305 Hz (experiment 2), and subsequently resampled offline at a sampling rate of 256 Hz. Signal conversion was performed using custom-written Matlab (The MathWorks Inc., Natick, MA, USA) scripts. Signals were then transformed into European Data Format (EDF). For each recording, EEG power spectra were computed by a fast Fourier transform (FFT) routine for 4-s epochs (Hanning window), with a 0.25-Hz resolution (SleepSign Kissei Comtec Co, Nagano, Japan). All spectral analysis was based on signals acquired from the frontal EEG derivation, with the exception of one individual mouse in experiment 1, in which the occipital derivation was used, because the frontal signal was lost for technical reasons.

### Statistical analysis

All data are expressed as mean ± standard error of the mean. Statistical analysis was performed using the Matlab (The MathWorks Inc., Natick, MA, USA) Statistics and Machine Learning Toolbox and custom-written Matlab scripts. Wilcoxon tests were applied for comparing effects between any two experimental conditions without an additional time domain. Normality was estimated by the use of the Anderson-Darling Test for Normality. Datasets with an additional time domain were tested with repeated measures ANOVA or mixed-model ANOVA (Laurent Caplette, MATLAB Central File Exchange) and, if required, followed up with Tukey post hoc tests for specific comparisons between the groups. The level for statistical significance was set to *p* < 0.05. In experiment 1, some animals were excluded from specific analyses, either because they did not perform one of the conditions for technical reasons, or because EEG data were available from one of the two derivations only. In such cases, we used unpaired Wilcoxon tests to compare groups, and paired Wilcoxon tests when comparisons were made within an animal. The number of animals and the type of test used are mentioned in corresponding figure legends as appropriate.

### Scoring and analysis of vigilance states

Vigilance states were scored offline through manual visual inspection of consecutive 4-s epochs (SleepSign, Kissei Comtec Co, Nagano, Japan). Two EEG channels (frontal and occipital) and EMG were displayed simultaneously to aid vigilance state scoring. Conventionally used criteria for vigilance state annotation were applied [60, 61]. Vigilance states were classified as waking (low-voltage, high-frequency EEG with a high level or phasic EMG activity), NREM sleep (presence of slow waves, a signal of a high amplitude and low frequency particularly in the frontal EEG derivation) or REM sleep (low-voltage, high-frequency EEG dominated by theta frequency activity, with a low level of EMG activity, particularly in the occipital EEG derivation). Epochs contaminated by eating, drinking or gross movements resulting in artefacts in at least one of the two EEG derivations were excluded from analyses. As the same experimenters obtained, processed and analysed the data, blinding to the animals’ identities was not always possible.

### Sleep deprivation

Animals were sleep-deprived by manually introducing novel objects into their home cages at irregular intervals. This paradigm has previously been shown to result in an increase of homeostatic sleep pressure reflected in EEG slow-wave activity [27, 34, 61], especially when it is associated with exploratory behaviour [62]. Novel objects included nesting material, small wooden blocks and similar objects commonly used as environmental enrichment. We made use of the natural tendency of mice to explore novel objects to keep them maximally engaged and active (Additional file 3: Video V2). The animals were continuously monitored by the experimenter and novel objects were provided whenever an animal showed behavioural signs of drowsiness (immobility) or intrusion of delta activity in the EEG (in the case of experiment 1, where EEG was continuously recorded during all conditions). Sleep deprivation was undertaken for durations between approximately 1–6 h depending on the experimental paradigm (see below).


**Additional file 3: Video V2.** Supplementary Video V2. Exploratory wakefulness (EW) in the touch screen chamber. Novel objects (as shown here) are provided to the mouse by the experimenter. Objects are changed or added whenever the animal shows behavioural quiescence.

### Experiment 1: The effects of voluntary wheel running on sleep

Running wheel (RW) activity is a widely used behavioural assay, utilising the well-described tendency of mice to spontaneously run in wheels when they are provided [11, 63]. Animals had free access to running wheels (Campden Instruments, Loughborough, UK, wheel diameter 14 cm, bars spaced 1.11 cm apart (inclusive of bars)) in their home cages for at least four weeks prior to the analysed dark period until ZT9 on each experimental day (Fig. 1) and were therefore well adapted to the wheels. The wheels were custom made for tethered animals, and tethering did not prevent the animals from running ad libitum (Fig. 2a). Each running wheel was fitted with a digital counter (Campden Instruments), which uses an infra-red (IR) emitter/receiver to detect each rung passing the IR beam as the wheel rotates. In our study, RW activity was recorded with a high temporal resolution within the same system used to record electrophysiology signals, as reported previously [5]. One full wheel revolution consisted of 38 individually detected rung counts, thus 10 counts per second corresponds to a speed of 10.11 cm/s. The wheel counter output was a 5 V TTL pulse (0 V with no output) that triggered an edge detector in the TDT acquisition system, and in turn created a timestamp that was stored for each wheel count.

After habituation to the chamber, baseline recordings of undisturbed sleep and wakefulness spanning the 24 h immediately before the experimental manipulation were performed. On the experimental day, all animals were sleep deprived (SD) for the last 3 h of the light phase without access to the running wheels and then subsequently kept awake for a further 3 h from dark onset either by the introduction of novel objects to elicit predominantly exploratory behaviour (exploratory wakefulness (EW), *n* = 8), or by providing access to the wheels (RW, *n* = 6), which the mice used extensively (Fig. 1). At ZT15, 3 h after dark onset, the novel objects were removed in the EW condition, and running wheels were blocked in the RW condition. All animals were then left undisturbed without wheel access for the rest of the dark period and could sleep ad libitum.

This experiment was performed at the end of the light period in order for the animals to efficiently build up sleep pressure (by keeping them awake for the last 3 h of their circadian rest phase) while ensuring voluntary wakefulness (either wheel running or exploratory behaviour) in the second half of the intervention, which took place at the beginning of their circadian active phase. Mice underwent both the RW and EW experimental conditions on separate days, though two mice were not recorded in the RW condition due to technical reasons (RW condition *n* = 6 mice, EW condition *n* = 8 mice).

### Experiment 2: The effects of touchscreen performance on sleep

To test the effects of voluntary engagement in an operant behaviour on sleep, we trained a separate group of adult male C57BL/6 mice (*n* = 5) using the Bussey-Saksida Touch Screen system [64, 65]. Our paradigm (called TS task) was designed specifically to allow for repetitive, well-trained behavioural sequences to occur (Additional file 2: Video V1). Mice were first habituated to the training chamber and the milkshake (Yazoo strawberry milk drink) reward that was delivered to the reward tray at the rear of the chamber. Throughout the experiment, animals underwent food restriction to maintain their body weight at approximately 85–90% of their average body weight during ad libitum food access. Mice were fed daily after dark onset (between ZT12 and ZT15) so that feeding-related wakefulness [2] would coincide with their circadian active period.

In the TS task, a trial consisted of nose-poking into a reward tray, followed by a single touch to an illuminated screen located in the centre of the opposite wall of the chamber, which initiated the next trial. After completion of every trial, or, in later training stages, of every third trial, the milkshake reward (0.0035 ml) was delivered to the reward tray. To encourage prolonged voluntary performance, there was no time limit for making a response in any part of the task. Note that trained animals still showed uniform response latencies across trials (Additional file 1: Figure S3C). Daily training sessions were conducted over 2–4 weeks, typically lasting 30–45 min per day depending on how long it took for each mouse to complete at least 100 trials.

Mice were then tested in the two conditions of the experiment on two separate experimental days. On the first experimental day, the animals were disconnected and placed into the touchscreen chamber at ZT0 (light onset) and allowed to perform the TS task continuously until they stopped engaging in the task for ten consecutive minutes, at which point they were transferred back into their home cage and reconnected to the recording setup. Pilot data from our lab suggested that animals may perform the task continuously for up to 7 h or even longer (data not shown). In the current study, animals performed for an average of 2.5 h. The experiment was conducted at the beginning of the light period as this is typically the start of the rest phase of mice. Performance in the task is therefore an effective form of sleep deprivation even for short task durations. Three to four days after the first experimental day, the animals were again transferred into the touchscreen chambers at light onset and kept awake by the introduction of novel objects, to induce exploratory wakefulness (EW), for the same amount of time as they had previously performed the TS task. Here, the environment and the time spent awake were matched between the two conditions, i.e. the duration of the voluntary performance in the TS task determined the duration of the sleep deprivation in the EW condition. This experimental design prevented us from counterbalancing the order of TS and EW experimental days. To control for the effects of food restriction and feeding on sleep [2], each mouse in the EW condition was provided with the same type and volume of milkshake as in the previous TS task condition, administered at regular intervals throughout the EW period. Continuous EEG and EMG recordings were conducted throughout the experiment, starting at least one day prior to the first experimental day and continuing until 1 day after the last experimental day. No electrophysiological recordings were obtained during engagement in either behavioural manipulation on the experimental days so as not to interfere with behavioural performance. Animals did not have access to running wheels during this experiment.

## Supplementary Information

**Additional file 1: Figure S1.** Wake EEG power spectra after and before EW and RW. Relative wake EEG power spectra during the first 15 min after EW/RW as compared to the 15-min interval immediately preceding EW/RW. Mean values, SEM. The horizontal lines below the curves denote frequency bins where EEG power showed a systematic change (black: *p*<0.05, grey: *p*<0.1, Wilcoxon rank sum test) in RW (top line), EW (middle line) or differed between EW and RW conditions (bottom). **Figure S2.** Time course of sleep after RW/EW wakefulness. Data depict total sleep amount during the first 6 h after the animals were left undisturbed after RW and EW conditions (mean values, SEM). **Figure S3.** Voluntary wakefulness during an operant task. (A) Schematic of the operant task environment. A trial consists of nose-poking into a reward tray (1), followed by a single touch to an illuminated screen located in the centre of the opposite wall of the chamber (2). After completion of every three trials a food reward is administered into the reward tray. Collecting the food reward initiates the next trial (3). (B) Behavioural results of daily operant training depicted as completed trials per session. (C) Behaviour during condition 1 (ad libitum performance in the TS task). Left: Behaviour shown as trial counts per 5 min bin for each animal. Right: Behaviour depicted as latencies between touches to any screen or the food tray. Colours depict individual animals. Lengths of vertical lines depict latencies, x values of vertical lines depict onset of each measured latency (i.e. the last touch event). Star for animal 4 (green) depicts inactive time that was accounted for in the EW condition (i.e. the mouse was allowed to sleep after 3 hours). **Figure S4.** Effects of TS and EW tasks on EEG SWA in individual animals. Plots show the same data as depicted in Fig. 3D. Dots signify individual animals. Lines between dots connect data points of the same animal in TS and EW conditions. **Figure S5.** Wake EEG spectra after as compared to before TS/EW conditions. (A) Wake EEG power spectra during 60 min. after TS/EW conditions relative to wake EEG spectra calculated over a window of 60 min. immediately before TS/EW in the frontal derivation. (B) Data depicted as in panel A but for the occipital derivation. Mean values, SEM, *n*=5.

## Data Availability

Datasets used and/or analysed during the current study are presented in the main paper or, where applicable, will be made available from the corresponding author on request.
