# Supplementary figures and images for: Waking experience modulates sleep need in mice
Source: BMC Biol. 2021 Apr 6;19:65. doi: 10.1186/s12915-021-00982-w (PMC8025572; doi:10.1186/s12915-021-00982-w)

## Slide 1
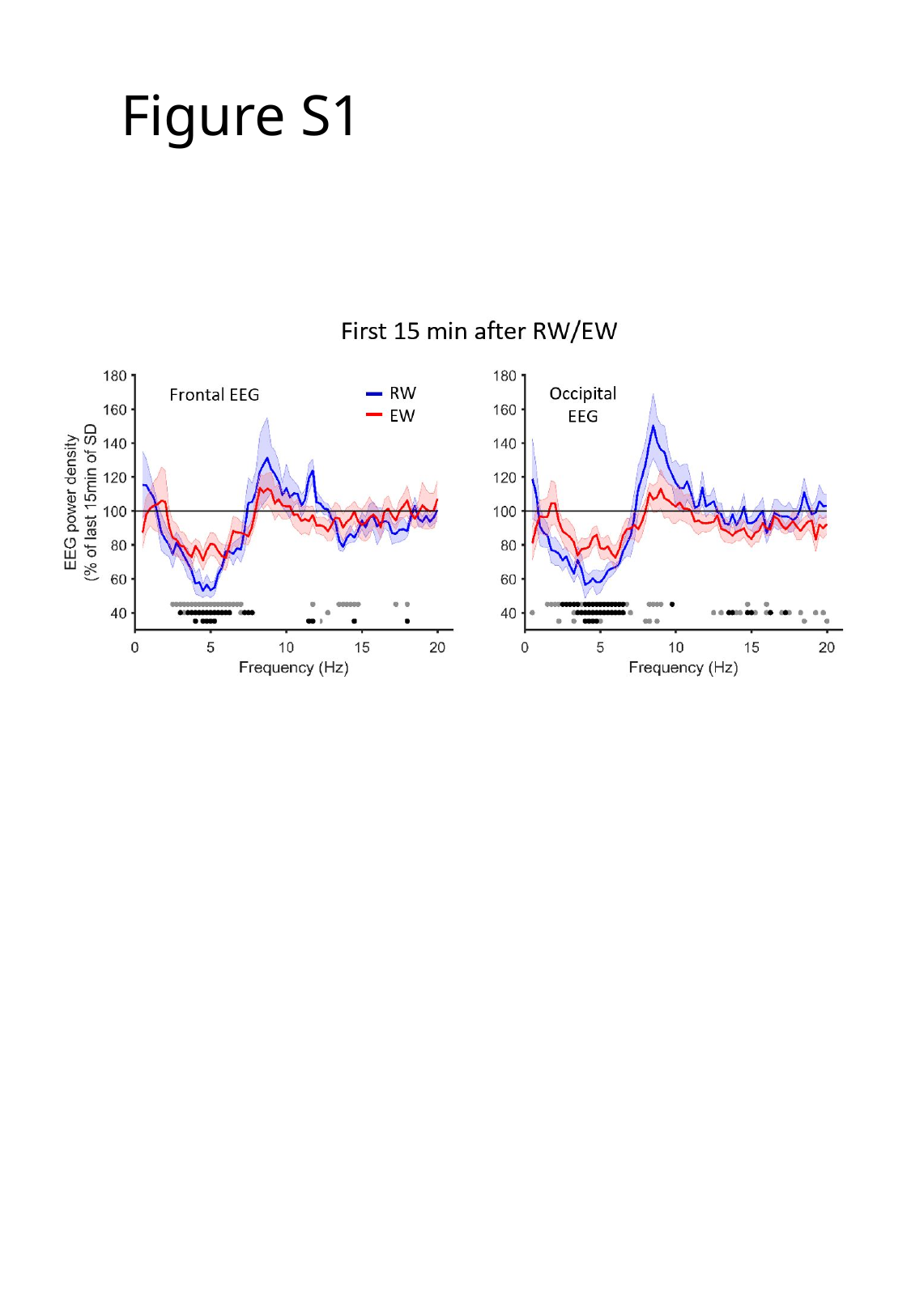

Figure S1

## Slide 2
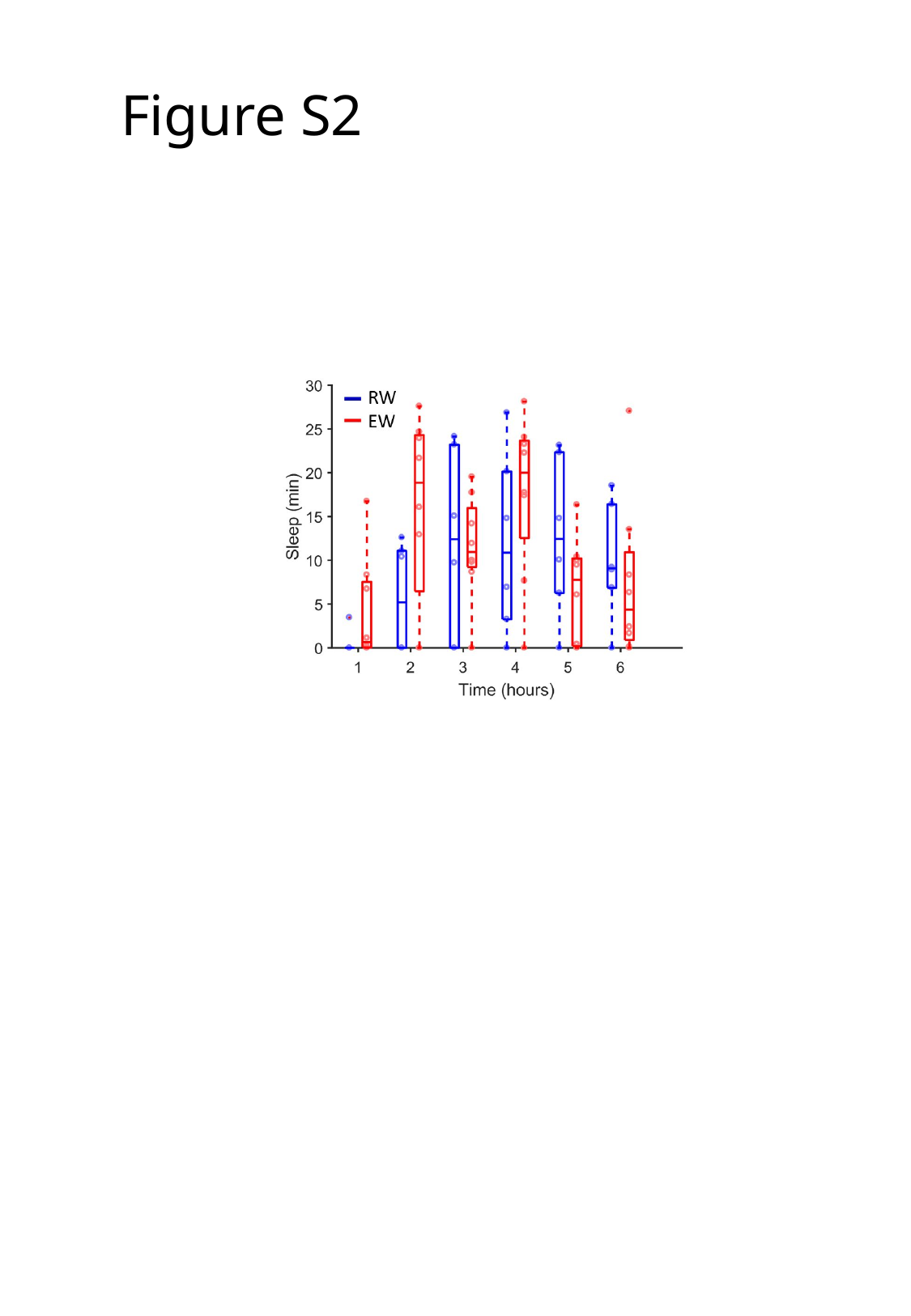

Figure S2

## Slide 3
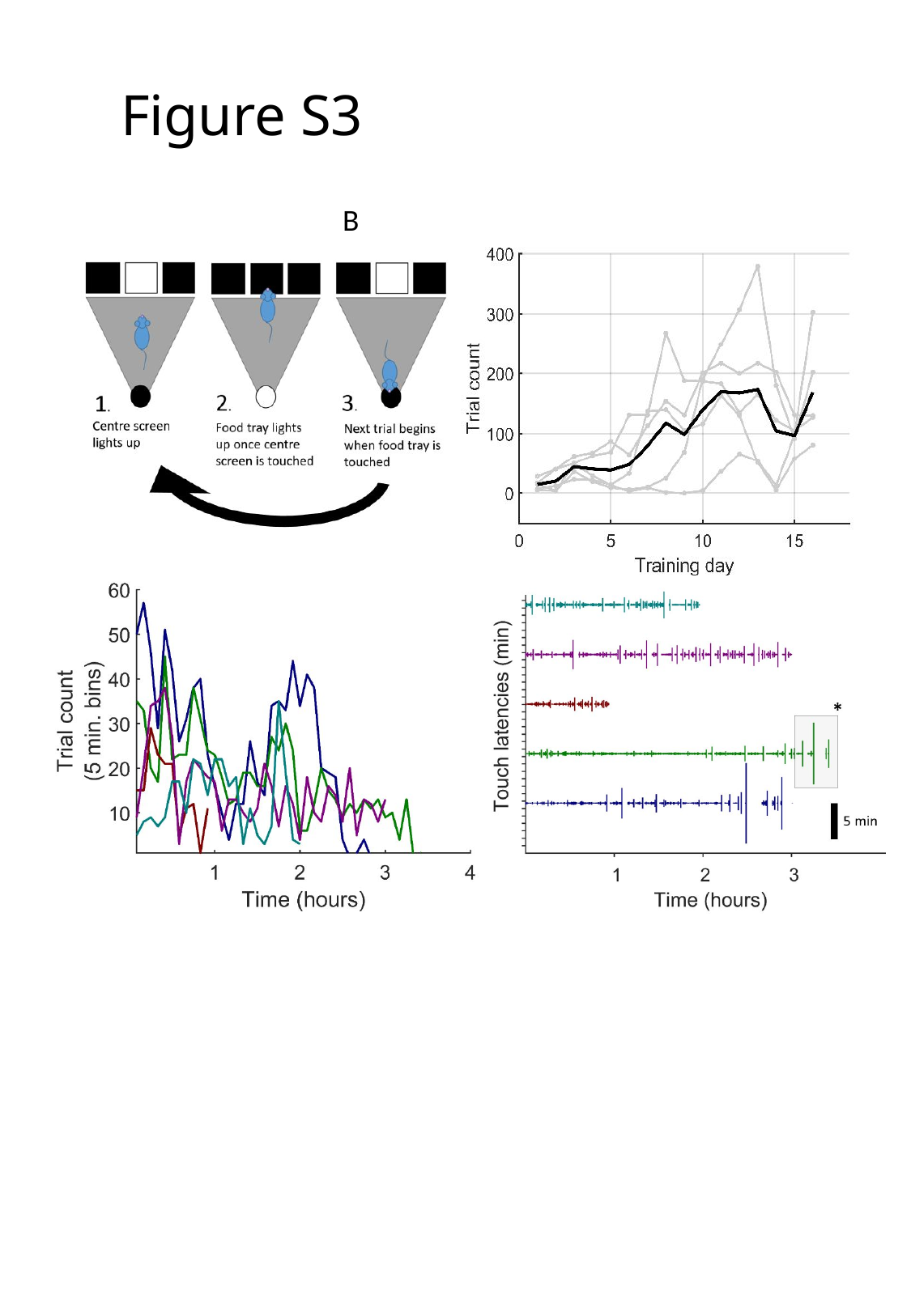

Figure S3
A B
C

## Slide 4
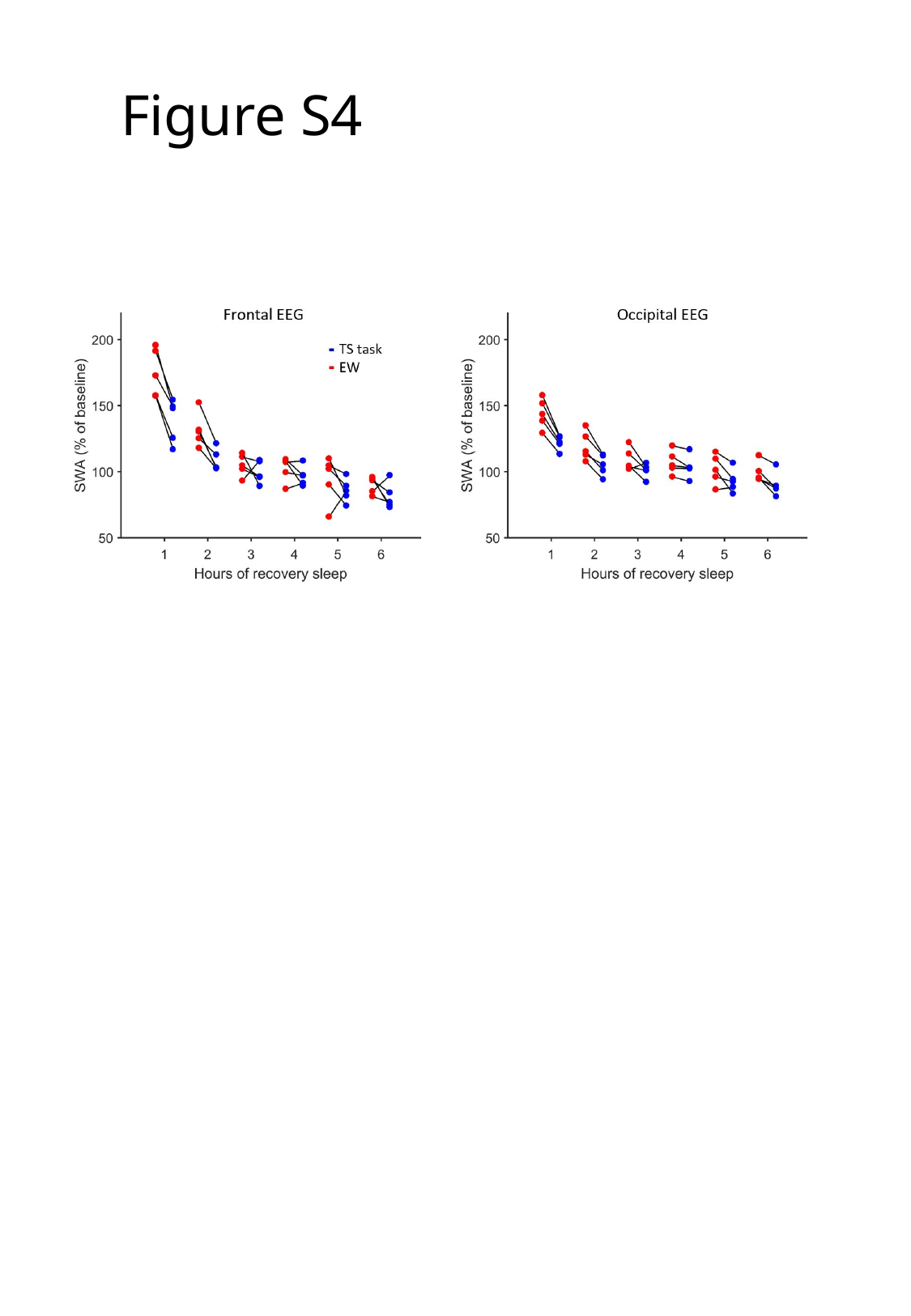

Figure S4

## Slide 5
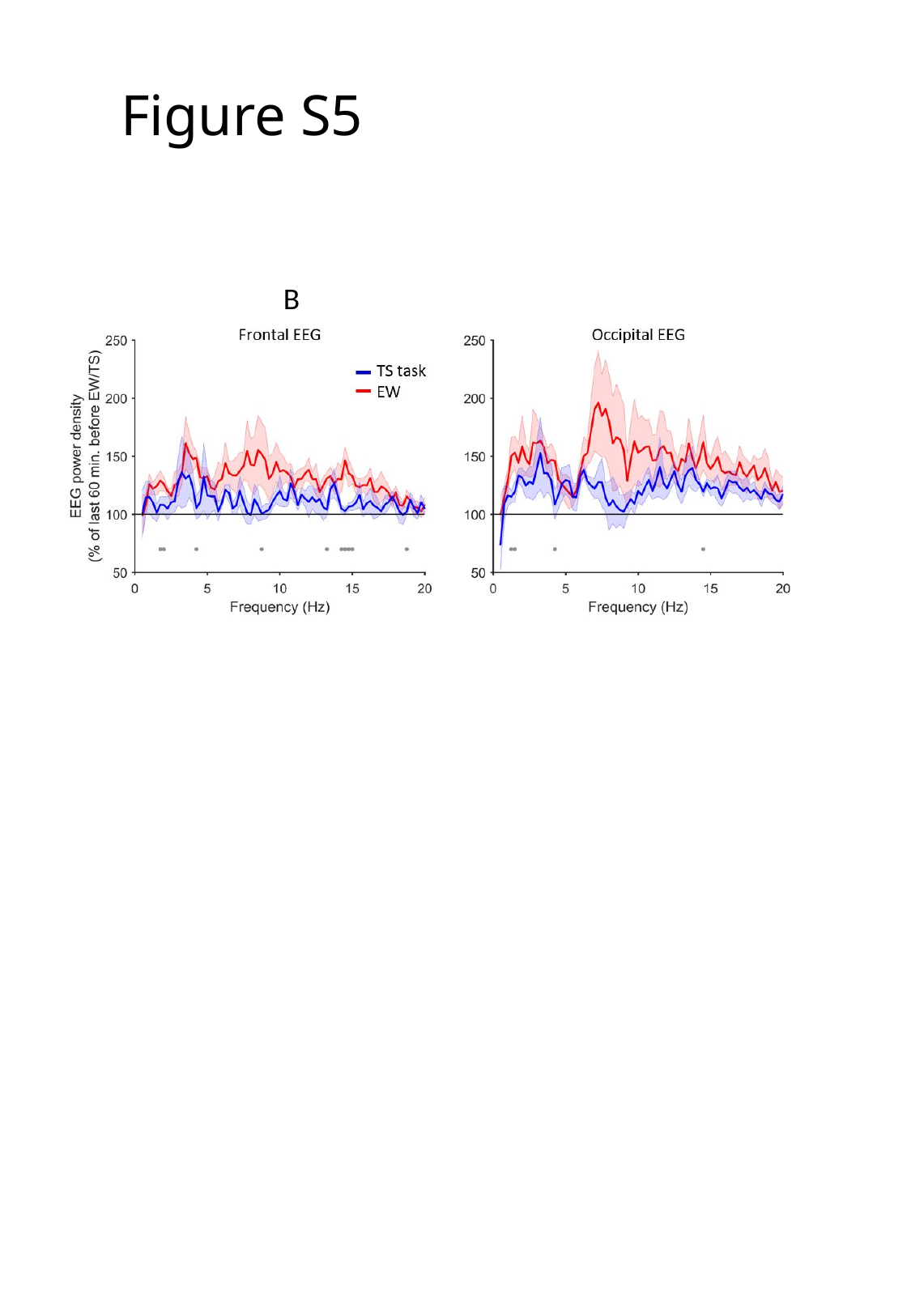

Figure S5
A B

Supplement: Supplementary file 3 — Additional file 1: Figure S1. Wake EEG power spectra after and before EW and RW. Relative wake EEG power spectra during the first 15 min after EW/RW as compared to the 15-min interval immediately preceding EW/RW. Mean values, SEM. The horizontal lines below the curves denote frequency bins where EEG power showed a systematic change (black: p<0.05, grey: p<0.1, Wilcoxon rank sum test) in RW (top line), EW (middle line) or differed between EW and RW conditions (bottom). Figure S2. Time course of sleep after RW/EW wakefulness. Data depict total sleep amount during the first 6 h after the animals were left undisturbed after RW and EW conditions (mean values, SEM). Figure S3. Voluntary wakefulness during an operant task. (A) Schematic of the operant task environment. A trial consists of nose-poking into a reward tray (1), followed by a single touch to an illuminated screen located in the centre of the opposite wall of the chamber (2). After completion of every three trials a food reward is administered into the reward tray. Collecting the food reward initiates the next trial (3). (B) Behavioural results of daily operant training depicted as completed trials per session. (C) Behaviour during condition 1 (ad libitum performance in the TS task). Left: Behaviour shown as trial counts per 5 min bin for each animal. Right: Behaviour depicted as latencies between touches to any screen or the food tray. Colours depict individual animals. Lengths of vertical lines depict latencies, x values of vertical lines depict onset of each measured latency (i.e. the last touch event). Star for animal 4 (green) depicts inactive time that was accounted for in the EW condition (i.e. the mouse was allowed to sleep after 3 hours). Figure S4. Effects of TS and EW tasks on EEG SWA in individual animals. Plots show the same data as depicted in Fig. 3D. Dots signify individual animals. Lines between dots connect data points of the same animal in TS and EW conditions. Figure S5. Wake EEG spectra afte [file 12915_2021_982_MOESM1_ESM.pptx]
